# Retrospective Evaluation of Dual Specialty Ports in Therapeutic Apheresis

**DOI:** 10.1002/jca.70126

**Published:** 2026-04-29

**Authors:** Mugtaba Swar‐Eldahab, Barnabas Obeng‐Gyasi, Paul Haste, Matthew Krosin

**Affiliations:** ^1^ Department of Radiology and Imaging Sciences Indiana University School of Medicine Indianapolis Indiana USA

**Keywords:** clinical outcomes, implantable ports, port complications, PowerFlow technology, quality of life, therapeutic apheresis, vascular access devices

## Abstract

Dual specialty ports were evaluated for safety and efficacy in therapeutic apheresis by analyzing outcomes across 97 port placement events in 88 patients, focusing on two configurations: dual Bard PowerFlow (BP2) and a combination of Bard PowerFlow with AngioDynamics SmartPort (BP + AD). This retrospective cohort study was conducted at a tertiary care center from December 2017 to July 2024 and included 97 port placement events (194 total ports) across 88 patients with conditions such as bronchiolitis obliterans, sickle cell disease, myasthenia gravis, and graft‐versus‐host disease who required therapeutic apheresis. Measured outcomes included port revisions, removals, total port days, and complications. The BP2 configuration was used in 43 events (44.3%), and the BP + AD configuration in 54 events (55.7%). Common indications included bronchiolitis obliterans (40.2%), sickle cell disease (17.5%), myasthenia gravis (12.4%), and GVHD (12.4%). Revisions were necessary in 5.7% of ports due to mechanical issues or thrombosis, while 36.1% of ports were removed, mainly due to infection/bacteremia or completion of therapy. Port functionality ranged from 22 to 2080 days, with a mean of 564 days. The mortality rate during follow‐up was 21.6%. Dual specialty ports demonstrate viable long‐term access for therapeutic apheresis in selected patients, with functionality extending from months to years. Success appears highly dependent on patient selection, with infection risks particularly notable in immunocompromised populations. These findings support the use of specialty ports as an alternative to traditional tunneled catheters, particularly for those with preserved immune function requiring chronic therapy.

**Abbreviations**
AD: AngioDynamics SmartPort
BP: Bard PowerFlow
CIDP: chronic inflammatory demyelinating polyneuropathy
CLABSI: catheter‐related bloodstream infection
CVC: central venous catheter
Dual PowerFlow configuration: PowerFlow plus AngioDynamics SmartPort configuration
GVHD: graft‐versus‐host disease

## 1 Introduction

Therapeutic apheresis, a procedure that selectively removes harmful components from the blood before returning it to the patient's circulation, plays a pivotal role in managing severe medical conditions [1, 2]. This treatment modality is essential for conditions such as bronchiolitis obliterans, myasthenia gravis, graft‐versus‐host disease (GVHD), and sickle cell disease (requiring red cell exchange), which often require repeated sessions over extended periods.

Conventionally, apheresis is performed through large‐bore tunneled or temporary catheters [3]. However, these external devices can significantly impact quality of life, with limitations on showering and physical activity, while also presenting cosmetic concerns for patients requiring long‐term therapy [4].

More recently, certain centers have begun utilizing implanted specialty ports in select patients with chronic and established need for apheresis. These fully implanted devices offer potential advantages in patient comfort and lifestyle considerations. At our institution, two primary port configurations have been employed: the dual PowerFlow (BP2) configuration utilizing two Bard PowerFlow Implantable Ports (BD, Franklin Lakes, NJ) or the combination (BP + AD) configuration using one Bard PowerFlow Port and one AngioDynamics SmartPort with Vortex Technology (AngioDynamics, Latham, NY). While these implanted ports may offer advantages for long‐term apheresis therapy, they require careful consideration of potential complications including infection, mechanical malfunction requiring revision, and thrombosis. The decision to place, remove, or revise a port depends on multiple factors, ranging from the expected duration of therapy to complications necessitating intervention [5].

This study evaluates the safety and efficacy of dual specialty ports for therapeutic apheresis, addressing an important gap in the literature regarding their real‐world performance as alternatives to traditional large‐bore catheters. Our findings aim to guide clinical decision making for long‐term apheresis therapy and identify which patient populations may benefit most from implanted specialty ports over conventional access methods.

Although single‐needle apheresis is technically feasible for the procedures included in this study, our institution elected to utilize dual port configurations based on both clinical and operational considerations. Dual port access allows for higher flow rates and more efficient completion of apheresis sessions, which is particularly advantageous in high‐volume centers where reduced procedure duration facilitates greater daily patient capacity. This approach was developed collaboratively with the apheresis providers to optimize workflow while maintaining procedural safety and treatment efficacy.

## 2 Materials and Methods

### 2.1 Study Design and Setting

This retrospective cohort study was conducted at a tertiary care center to evaluate the safety, efficacy, and complication rates of two configurations of specialty ports used in therapeutic apheresis: dual Bard PowerFlow Implantable Apheresis IV Ports (BP2; BD, Franklin Lakes, NJ) or a combination of one BP and one AngioDynamics SmartPort with Vortex Technology (AD; AngioDynamics, Latham, NY). The study period spanned from December 2017 to July 2024.

### 2.2 Participants

A total of 97 port placement events across 88 patients were analyzed. Each placement event represented either the initial simultaneous placement of two ports (dual BP or BP + AD configuration) or the replacement of an existing configuration. Patients included in the study required therapeutic apheresis for various medical conditions including bronchiolitis obliterans, myasthenia gravis, graft‐versus‐host disease (GVHD), and red blood cell exchange for sickle cell disease. In some cases, patients had multiple placement events over the study period when ports were removed after completing a course of therapy but later required new ports due to disease recurrence.

Selection of port configuration was based on anticipated clinical needs and access requirements at the time of placement. Patients with expected frequent non‐apheresis access needs, such as those with sickle cell disease requiring recurrent hospitalizations, pain management admissions, or frequent laboratory evaluation, were preferentially assigned the BP + AD configuration. This provided access through the AD port using a standard Huber needle, which is familiar to inpatient units, emergency departments, and infusion centers, in contrast to the BP port, which requires access with a 16G angiocatheter and is typically limited to apheresis‐trained staff.

Patients undergoing extracorporeal photopheresis or plasma exchange requiring prolonged sessions and higher flow rates were more commonly assigned the BP2 configuration to facilitate more efficient apheresis treatments and reduce session duration. While photopheresis and plasma exchange can be performed using either configuration, BP2 access was favored when minimizing treatment time was a priority in patients without anticipated need for frequent general venous access.

In the BP + AD configuration, the BP port was intended for blood extraction and the AD port for return. This access strategy was established early during the development of the apheresis port service line in collaboration with apheresis providers at our institution and applied consistently during port placement. All port placements were performed within a single practice by a limited group of interventional radiologists, allowing this convention to be applied uniformly, including during catheter length selection and tip offset to minimize recirculation.

At the time of placement, catheter tip positions were intentionally staggered to minimize the risk of recirculation during apheresis. A standardized institutional convention was used in which the longer catheter was positioned laterally and the shorter catheter medially. This configuration allowed blood extraction from the medial port with return through the lateral port as a consistent practice within the apheresis unit.

This convention was applied to both BP2 and BP + AD configurations. In the BP + AD configuration, the lateral port was preferentially designated as the AD port, allowing patients to be counseled that the lateral port, located closer to the shoulder, could be used for general venous access in inpatient units, emergency departments, laboratories, or infusion centers. With this standardized approach to catheter tip positioning and port designation, reversal of access and return ports due to sluggish flow or pressure alarms was not routinely required.

Locking solutions and maintenance strategies differed by port type and were used in accordance with manufacturer recommendations. The AD ports were routinely locked with 10 units/mL heparin, whereas the BP ports were locked with 1000 units/mL heparin. In patients with contraindications to heparin, alternative locking solutions such as sodium bicarbonate were used, although this occurred infrequently.

Flow rates during therapeutic apheresis varied based on the procedure being performed. Most placements in this cohort were used for red blood cell exchange or extracorporeal photopheresis, which at our institution typically operate at maximum inlet flow rates of approximately 50 mL/min.

For plasma exchange and red blood cell exchange procedures, single‐needle mode was not available at our institution. In cases where one port demonstrated inadequate flow, initial management consisted of reaccessing the port when appropriate. If adequate flow could not be restored, peripheral intravenous access was obtained with assistance from the vascular access team when feasible, and procedures were infrequently shortened or aborted.

In contrast, for extracorporeal photopheresis treatments, single‐needle mode was available and used when one port remained fully functional. Management in these cases included reaccessing the port when appropriate and instillation of tPA into the nonfunctional port. When possible, peripheral intravenous access was placed to allow a return to double‐needle mode. In most cases, impaired flow was suspected to be related to fibrin sheath formation, as flow typically improved following tPA administration.

### 2.3 Data Collection

Initial case identification was performed using an internal radiology information system search engine to identify relevant procedures. Detailed clinical data were then extracted from the electronic health record. Port configurations were categorized as either dual BP2 (two Bard PowerFlow ports) or BP + AD (one Bard PowerFlow port plus one AngioDynamics SmartPort). Information collected included patient demographics, primary diagnosis, indications for apheresis, and clinical outcomes including port revisions, removals, and complications.

### 2.4 Outcome Measures

The primary outcomes measured were port revisions and removals, categorized by reason for intervention (e.g., no longer needed, infection, mechanical malfunction). For the purposes of this study, a port revision was defined as any procedure in which an existing port was modified or replaced, and a new port was left in place at the conclusion of the procedure, whether of the same or a different configuration. Indications for revision included mechanical malfunction, fibrin sheath formation, difficulty with access due to patient habitus, or port rotation or flipping. In contrast, port removal referred to procedures in which ports were completely explanted without immediate replacement, most commonly due to suspected infection or because the port was no longer required following completion of therapy.

Secondary outcomes included total port days, calculated from placement until removal or end of study period, to assess durability and long‐term usability of each configuration. For patients who died with ports in place, port days were calculated up to the date of death. While patient mortality was tracked, this was primarily to accurately calculate port days and understand the natural history of port use in this population, as deaths were predominantly related to progression of underlying disease.

### 2.5 Statistical Analysis

Descriptive statistics were used to summarize port types and clinical indications. The incidence of port interventions (removals and revisions) was calculated as frequencies and percentages. Statistical significance was set at a *p* value of less than 0.05. All analyses were performed using SAS software, version 9.4.

### 2.6 Ethical Considerations

The study protocol met institutional review board (IRB) exemption criteria at our institution. Due to the retrospective nature of the study and the use of de‐identified patient data, a waiver of informed consent was granted.

## 3 Results

### 3.1 Port Placement and Types

During the study period from December 2017 to July 2024, a total of 97 port placement events were recorded across 88 patients at the tertiary care center, resulting in 194 total ports placed. The configurations used were: dual BP (two Bard PowerFlow ports) in 43 events (44.3%, 86 total ports), and BP + AD configuration (one Bard PowerFlow port plus one AngioDynamics SmartPort) in 54 events (55.7%, 108 total ports) (Table 1).

**TABLE 1:** Patient and port characteristics.

Characteristic	Value
Study population	
Total port placement events	97
Unique patients	88
Port configuration	
BP2 configuration	43 (44.3%)
BP + AD configuration	54 (55.7%)
Primary diagnosis	
Bronchiolitis obliterans	39 (40.2%)
Sickle cell disease	17 (17.5%)
Myasthenia gravis	12 (12.4%)
GVHD	12 (12.4%)
Sezary syndrome	6 (6.2%)
Other^a^	11 (11.3%)
Interventions	
Port revisions	11 (11.3%)
Port removals	35 (36.1%)
Mortality	
Deaths during study period	19 (21.6%)

^a^ Other includes: CIDP (3), T‐Cell Lymphoma (2), Lambert‐Eaton syndrome (1), Mycosis fungoides (1), Paraneoplastic neuromyopathy (1), Waldenstrom's macroglobulinemia (1), Optic neuritis (1), and Myelodysplastic disorder (1).

### 3.2 Indications for Port Use

The primary indications for port placement varied by underlying condition. Bronchiolitis obliterans was the most common indication, accounting for 39 events (40.2%). These patients, along with those with Sezary syndrome (6.2%) and GVHD (12.4%), primarily underwent extracorporeal photopheresis (ECP), a specific type of therapeutic apheresis procedure, through the implanted specialty ports. Sickle cell disease patients requiring chronic red blood cell exchange therapy represented 17 events (17.5%). These patients benefited from the additional functionality of ports for routine blood draws and medication administration. Myasthenia gravis and graft‐versus‐host disease (GVHD) each accounted for 12 events (12.4%). The remaining indications included Sezary syndrome (6.2%) and other conditions (11.3%) (Tables 1 and 2).

**TABLE 2:** Outcomes by treatment type.

Characteristic	Value
RBC exchange^a^	
Patients (*N*)	17
Mean port days	729.8
Infection rate (%)	23.5
Removal rate (%)	29.4
Photopheresis	
Patients (*N*)	60
Mean port days	562.8
Infection rate (%)	20.0
Removal rate (%)	33.3
Plasma exchange	
Patients (*N*)	20
Mean port days	502.6
Infection rate (%)	25.0
Removal rate (%)	50.0

^a^ RBC exchange includes all sickle cell disease patients requiring chronic transfusion therapy. Infection rate calculated as number of ports removed for infection/bacteremia divided by total ports. Port days calculated from placement to removal or end of study period. Removal rate includes all causes of port removal including completion of therapy.

### 3.3 Port Revisions and Removals

Of the 194 total ports placed, 11 ports in 9 patients (5.7% of total ports) required revisions due to mechanical malfunction, thrombosis, or suboptimal performance (Tables 1 and 3). Port removals occurred in 70 ports (35 placement events, 36.1% of total ports). The primary reasons for removal included suspected infection or bacteremia (60.0%) and completion of therapy with ports no longer needed (34.3%) (Tables 4 and S5). In cases of suspected infection, both ports were typically removed as a precautionary measure, particularly in bacteremic patients, though isolated port site infections occasionally resulted in single port removal.

**TABLE 3:** Comparison of outcomes between port configurations.

Characteristic	BP2 configuration (*n* = 43)	BP + AD configuration (*n* = 54)	*p*
Port revisions	6 (14.0%)	5 (9.3%)	0.469
Port removals	18 (41.9%)	17 (31.5%)	0.290
Mortality	10 (23.3%)	9 (16.7%)	0.417
Port days			
Mean ± SD	496.0 ± 576.3	628.9 ± 545.4	0.324^a^
Median	243.5	522.0	

^a^ *p* value calculated using Mann–Whitney *U* test due to non‐normal distribution of port days.

**TABLE 4:** Temporal analysis of port complications.

Characteristic	Early (< 30 days)	Late (> 30 days)
Infection^a^	3	18
Mechanical	0	0
No longer needed	0	12
Other	0	2

^a^ Early defined as ≤ 30 days from placement; late defined as > 30 days from placement. Infection includes bacteremia and local port site infections. Mechanical includes port malfunction and thrombosis. No longer needed includes completion of therapy and resolution of underlying condition. Other includes patient preference and non‐infectious complications.

### 3.4 Clinical Outcomes

The study observed a mortality rate of 21.6% (19 patients) during the follow‐up period (Table 1). All deaths were attributed to progression of underlying disease or unrelated medical complications. While one patient developed coagulase‐negative staphylococcus bacteremia (beyond 30 days post‐placement), this was likely related to the patient's underlying immunosuppressed state rather than directly attributable to initial port placement. This finding aligns with the known infection risks in immunocompromised patients with implanted devices, particularly those undergoing cancer treatment or post‐transplant care (Table 4).

Infection‐related outcomes were additionally analyzed by port configuration. Of the 21 infection‐related port removals, 11 occurred in the BP + AD configuration and 10 occurred in the BP2 configuration. The corresponding infection rates were 0.76 per 1000 port days for BP + AD and 0.92 per 1000 port days for BP2. Three early infections (< 30 days) were observed overall, with one occurring in the BP + AD configuration and two occurring in the BP2 configuration.

### 3.5 Port Durability

The duration of port functionality in patients meeting primary or secondary endpoints varied considerably, ranging from 22 to 2080 days, with a mean duration of 564 days and a median of 401 days (Table 3). This wide range reflects the diverse nature of underlying conditions and treatment requirements, with some patients requiring short‐term therapy while others maintained functional ports for several years (Table 2).

Previous bench studies have demonstrated that the PowerFlow implantable apheresis port can achieve flow rates up to 90 mL/min [6]. While we did not directly measure flow rates in our patient cohort, the high clinical success rate observed in our study confirms these earlier findings, indicating that specialty ports can achieve the necessary flow rates for effective apheresis treatment when properly selected and maintained [1]. This practical validation of flow adequacy is reflected in the extended functional duration and low complication rates observed across our patient population.

## 4 Discussion

Our retrospective evaluation of dual specialty ports in therapeutic apheresis suggests that these implantable devices are a safe and effective option for long‐term vascular access. The results demonstrate that specialty ports provide durable access with functionality extending from months to years, a finding that aligns with previous reports on implantable apheresis ports maintaining adequate blood flow rates for sustained therapy [6, 7]. Studies have shown that modern ports can sustain inlet flow rates sufficient for effective therapeutic apheresis when appropriately selected and maintained [8].

Patient selection remains a crucial factor in optimizing the benefits of specialty ports. In our institution, these devices were primarily placed in patients requiring prolonged or frequent apheresis treatments who lacked suitable peripheral venous access [9]. This approach aligns with broader recommendations suggesting that vascular access should be individualized based on treatment frequency, duration, and vascular anatomy [3]. Peripheral venous access is often preferred when feasible due to its lower procedural risk and avoidance of indwelling device complications [5]. However, for patients expected to undergo long‐term therapy, an implantable port can provide significant advantages, including reduced procedural burden during apheresis compared to use of peripheral IV access and improved patient comfort [10]. Apheresis centers implementing ports have reported similar selection criteria, prioritizing patients requiring extensive therapy, such as those undergoing chronic extracorporeal photopheresis [11]. Given these considerations, our study supports the paradigm that specialty ports should be reserved for patients who require ongoing apheresis and lack reliable peripheral venous access [12].

When analyzing infection‐related complications, our data revealed an infection rate of 0.83 per 1000 port days, which aligns with published literature citing rates of approximately 1 per 1000 days for implanted ports [9]. This is significantly lower than reported rates for traditional apheresis catheters, which exceed 6 per 1000 catheter days [9]. Among the 21 infection‐related port removals, the majority (19 cases) were due to bacteremia, with only 1 confirmed port site infection. Most infections (18 cases) occurred after 30 days of implantation, suggesting that these events may be related to repeated access rather than placement complications. Importantly, the attribution of bacteremia directly to port colonization versus removal as a precautionary measure in the setting of systemic infection remains challenging in our retrospective analysis. This is particularly relevant in our patient population, many of whom were immunocompromised due to underlying disease or therapy. Nevertheless, the observed infection rate supports the relative safety of specialty ports compared to external tunneled catheters for long‐term apheresis therapy [5].

Specialty ports provide several key advantages over traditional tunneled catheters, particularly for patients requiring chronic apheresis. These advantages include long‐term durability, adequate flow rates for effective therapy, and improved quality of life [11, 13]. In our cohort, we observed an infection rate of 0.83 per 1000 port days, which is within the range reported in literature for implantable ports. Unlike external catheters, ports allow patients to engage in normal activities, including bathing and exercise, without restriction [14, 15]. Additionally, maintenance requirements are significantly lower, with ports typically requiring only periodic heparin flushes which are performed during routine apheresis sessions, rather than frequent dressing changes [16]. Furthermore, early cost analyses by Williams LA 3rd, et al. suggest that ports may reduce overall healthcare expenditures by minimizing infection‐related hospitalizations and the need for frequent catheter replacements [3]. While large‐bore tunneled dialysis catheters are capable of substantially higher maximum flow rates, dual port configurations are designed to reliably support the flow rates required to successfully complete therapeutic apheresis procedures when appropriately selected and maintained.

Despite their benefits, specialty ports are not without limitations. The implantation procedure is more invasive than peripheral venous access, though comparable to tunneled line placement [6]. Potential complications aside from infection include thrombosis, catheter occlusion, bleeding, and mechanical failure, although these events are collectively uncommon and almost never major or life threatening [8]. Our study observed a small number of occlusion‐related revisions, consistent with previous findings that port‐related thrombosis is a manageable but real concern [1]. Device selection is also an important factor, as certain port configurations have been associated with more frequent flow alarms and access difficulties [3]. Additionally, training is required for healthcare providers unfamiliar with specialty port access techniques. While normal general ports including the SmartPort use standard Huber needles that most nurses are trained to use, the Bard PowerFlow apheresis ports require access with a 16G angiocatheter, necessitating special training for nurses, apheresis technicians, and even physicians who access them. Though apheresis ports can technically be used for general access, their non‐standard access method has led to our practice of discouraging their use outside of apheresis procedures [5]. Finally, while upfront costs for port placement are higher than for temporary or tunneled catheters, long‐term benefits such as potential reduction in hospital admissions and fewer replacements may justify the initial expense [3].

Our findings suggest that dual specialty ports represent a promising advancement in therapeutic apheresis access, offering a safe and durable alternative to traditional catheters [5]. The combination of long‐term usability and improved patient comfort makes these ports particularly valuable for patients requiring prolonged therapy [17]. Ongoing research and experience will continue to refine their role in clinical practice, ensuring that the benefits are maximized while potential limitations are mitigated through careful patient selection and device management [18].

## 5 Limitations

As a single‐center retrospective analysis, our findings may not be generalizable to all settings, and the lack of a direct comparator group limits our ability to draw definitive conclusions regarding superiority over traditional catheters. Selection bias is possible, as patients receiving ports were likely those anticipated to require long‐term therapy. Additionally, our follow‐up period may not have captured late‐onset complications such as device failure beyond the study timeframe. Despite these limitations, our study contributes to the growing body of evidence supporting the use of specialty ports in therapeutic apheresis (5). Future prospective studies should focus on direct comparisons between ports and other access methods, including tunneled dialysis catheters and arteriovenous fistulas, to further refine best practices in vascular access selection for apheresis patients.

## 6 Conclusion

This study demonstrates that specialty ports can provide durable long‐term access for therapeutic apheresis in carefully selected patients, with functionality extending from months to years. However, success appears highly dependent on patient selection, with our observed infection rate of 0.83 per 1000 port days highlighting particular risks in immunocompromised populations. While this rate falls within reported ranges for implantable ports in literature, infection remains a significant consideration. These findings support the role of specialty ports as a viable alternative to traditional tunneled catheters in appropriate candidates, particularly those with preserved immune function requiring chronic therapy.

## Author Contributions

**Mugtaba Swar‐Eldahab:** data collection, analysis, and manuscript drafting. **Barnabas Obeng‐Gyasi:** data collection, analysis, and manuscript drafting. **Paul Haste:** study design, data interpretation, and manuscript revision. **Matthew Krosin:** study conception and design, supervision, and critical revision.

## Funding

The authors have nothing to report.

## Disclosure

The authors have nothing to report.

## Ethics Statement

This retrospective study was conducted in accordance with the ethical standards of the institutional research committee and with the 1964 Helsinki Declaration and its later amendments. The study protocol met institutional review board (IRB) exemption criteria at Indiana University School of Medicine.

## Consent

Due to the retrospective nature of the study and the use of de‐identified patient data, a waiver of informed consent was granted by the institutional review board.

## Conflicts of Interest

The authors declare no conflicts of interest.

## Supporting information


**Data S1:** Detailed analysis of infection‐related outcomes and port days.

## Data Availability

The data that support the findings of this study are available from the corresponding author upon reasonable request, subject to institutional privacy and ethical considerations.
