# Supplementary material for: Retrospective Evaluation of Dual Specialty Ports in Therapeutic Apheresis
Source: J Clin Apher. 2026 Apr 29;41:e70126. doi: 10.1002/jca.70126 (PMC13127237; doi:10.1002/jca.70126)
Supplement: Supplementary file 1 — Data S1: Detailed analysis of infection‐related outcomes and port days. [file JCA-41-e70126-s001.docx]

Supplemental Table:

Table 5. Detailed Analysis of Infection-Related Outcomes and Port Days

| **Characteristic** |  | **Value** |
| --- | --- | --- |
| **Port Utilization** |  |  |
| Total placement events | 97 |  |
| Total ports placed | 194 |  |
| Total known port days | 25,378 | |
| **Removals** |  | |
| Total port removals | 35 (36.1%) | |
| **Infection-Related Outcomes** |  | |
| Infection-related removals | 21 (60.0% of removals) | |
| - Bacteremia | 19 (90.5% of infections) | |
| - Port site infection | 1 (4.8% of infections) | |
| - Other infection-related | 1 (4.8% of infections) | |
| Early infections (≤30 days) | 3 (14.3% of infections) | |
| Late infections (>30 days) | 18 (85.7% of infections) | |
| **Infection Rate** |  | |
| Per 1,000 port days | 0.83 | |

*Note: Early infections defined as ≤30 days from placement; Late infections defined as >30 days from placement. Infection rate calculated as (infection events / total port days) × 1,000. Port days calculated from placement to removal or end of study period. All percentages rounded to one decimal place.*
